# Human Supplementation with AM3, Spermidine, and Hesperidin Enhances Immune Function, Decreases Biological Age, and Improves Oxidative–Inflammatory State: A Randomized Controlled Trial

**DOI:** 10.3390/antiox13111391

**Published:** 2024-11-14

**Authors:** Judith Félix, Estefanía Díaz-Del Cerro, Adriana Baca, Ana López-Ballesteros, María José Gómez-Sánchez, Mónica De la Fuente

**Affiliations:** 1 Department of Genetics, Physiology and Microbiology (Animal Physiology Unit), Faculty of Biological Sciences, Complutense University of Madrid, 28040 Madrid, Spain; estedi01@ucm.es (E.D.-D.C.); adriabac@ucm.es (A.B.); 2 Institute of Investigation Hospital 12 Octubre (Imas12), 28041 Madrid, Spain; 3 Medical Affairs Department, Cantabria Labs, 28043 Madrid, Spain

**Keywords:** AM3, spermidine, hesperidin, immunity, biological age, redox state, inflammatory state

## Abstract

The positive effect of AM3, spermidine, and hesperidin, which have antioxidant and anti-inflammatory properties, on immunity is known, but their effect on the rate of aging, known as biological age (BA), is unclear. This work aims to test if the intake of a blend of AM3 (150 mg), spermidine (0.6 mg), and hesperidin (50 mg) for 2 months could decrease BA and improve immunity, redox, and inflammatory states. For this, 41 participants (30–63 years) were randomly divided into placebo and supplement groups. The supplement group took two capsules daily with AM3, spermidine, and hesperidin for two months, while the placebo group took capsules containing only calcium phosphate and talcum powder. Before and after the treatment, peripheral blood was collected. Immune function was assessed in leukocytes, redox state in whole-blood cells, erythrocytes, and plasma, and cytokine concentration in both mononuclear cell cultures and plasma. Finally, the Immunity Clock model was applied to determine BA. The results show that the intake of this blend improves the immune functions that constitute the Immunity Clock, decreasing BA by 11 years and reducing the oxidative–inflammatory state of the participants. Therefore, this supplement can be proposed as a strategy to rejuvenate BA and achieve healthy aging.

## 1. Introduction

Throughout the aging process, which begins in the twenties in humans, there is a progressive and generalized deterioration of the functions of an organism, especially in the homeostatic systems (nervous, endocrine, and immune), which leads to a loss of health and an increase in morbidity and mortality. This process is heterogeneous, so individuals of the same chronological age may have a different rate of aging or biological age, which is why the latter is a better marker of how aging is taking place and life expectancy [1].

Based on this, it has been considered that, of the homeostatic systems, the immune system is the most involved in the aging process, since it could participate in the establishment of the chronic oxidative and inflammatory stress that underly this process due to its ability to produce pro-oxidant and pro-inflammatory compounds [1,2]. Therefore, the immune system, which has been proposed as a good marker of health, has also been considered a marker of the rate of aging and a predictor of longevity [1,2,3]. This led to the development of a mathematical model for the determination of biological age, the Immunity Clock, based on different immune functions that are altered throughout the aging process, such as chemotactic and phagocytic capacity, natural killer activity, or the lymphoproliferative response to mitogens [4].

Given the frequent possibility that a person is aging faster than his or her chronological age and the risk of morbidity that this accelerated aging implies, ongoing research is looking for lifestyle strategies to slow down the rate of aging, and nutritional interventions are the most studied ones. Indeed, the intake of compounds with antioxidant and anti-inflammatory activities has been shown to be able to improve immune function [5,6], and decrease biological age [4].

In this context, AM3, the active principle of Immunoferon^®^, has been shown to have significant effects as a modulator of the immune response, with a clear regulatory role in inflammation [7,8,9,10,11,12,13,14,15,16,17,18,19,20,21,22]. However, its possible role in immunosenescence and oxidative stress has scarcely been studied [23,24], and, consequently, its possible usefulness in rejuvenating biological age is unknown.

In addition, polyamines, such as spermidine, are involved in a series of biological processes, mainly related to cell viability, proliferation, and differentiation. In fact, polyamine concentrations are decreased in many pathological states and aging, and their administration has been proposed to reduce disease risk. This is because they show antioxidant and anti-inflammatory properties, managing the redox state and inflammation of cells. Thus, it has become evident that they are relevant for maintaining health and achieving healthy aging as well as longevity [25,26,27,28,29,30,31,32,33,34,35,36,37,38,39,40,41].

Finally, flavonoids such as hesperidin are bioactive substances found mainly in fruits and vegetables [42]. Previous studies have shown that hesperidin has several pharmacological activities, suggesting that they are of great utility in the treatment of different diseases [43,44,45,46,47,48,49,50]. These effects may be due to its antioxidant and anti-inflammatory properties [51,52], as it inhibits the secretion of pro-inflammatory cytokines [53,54,55,56] and modulates leukocyte gene expression by enhancing its antioxidant and inflammatory profiles [57,58].

Based on all of the above, there is still a lack of knowledge on whether the ingestion of a blend containing these three compounds (AM3, spermidine, and hesperidin) can have a positive effect on immunity, oxidative and inflammatory state, and the biological age of men and women throughout their aging processes. Therefore, the aim of the present study was to test if daily supplementation for two months with AM3, spermidine, and hesperidin allows the biological age of healthy individuals to be decreased by improving their immunity and oxidative–inflammatory state.

## 2. Materials and Methods

### 2.1. Participants, Study Design, and Extraction of Blood Samples

This prospective, randomized, and double-blind trial was conducted at Universidad Complutense de Madrid (Madrid, Spain). All participants provided written informed consent, and the study was approved by the Ethical Committee of the Hospital Clínico San Carlos of Madrid (Madrid, Spain) (I.C. 22/065-EC_X). Clinical trial registry: NCT06249620 (https://clinicaltrials.gov accessed on 2 August 2024)

The participants in this study were 41 participants aged between 30 and 60 years. The participants were randomly divided into 2 experimental groups: the placebo group (N = 18; 7 men and 11 women) and the supplement group (N = 23; 11 men and 12 women). However, 6 participants were excluded from the study because they did not follow the treatment, which left the following groups: placebo (N = 15; 7 men and 8 women), supplement (N = 20; 10 men and 10 women). The sample size was calculated based on the number of groups in the study, the maximum number of groups in simultaneous comparison, and by assuming a significance level of the bilateral tests to be performed equal to 0.05, a required power of the test equal to 0.8, and assuming a standard deviation of 1, to obtain a minimum difference to be detected equal to 1.5.

The study population comprised healthy individuals, defined as those free from any clinically significant illnesses or abnormalities in routine laboratory evaluations. The exclusion criteria encompassed individuals with significant health conditions, including autoimmune disorders, cancer, anemia, severe allergies, dementia or cognitive impairments, chronic respiratory illnesses, hypertension, and diabetes. Additional exclusions were applied to individuals with a history of alcohol or drug use, those undergoing hormone replacement therapy, and those taking vitamins, antioxidants, or any medications with potential immunomodulatory effects. Participants with poor compliance with study protocols were also excluded. Informed consent was obtained from all participants, allowing for the use of their blood samples in research.

Before starting the treatment, quality of life was assessed with a survey that included the Perceived Stress Scale (PSS), the Hamilton Anxiety Rating Scale (HAM-A), and the 14-Item Resilience Scale (RS-14). It also included yes/no questions about whether they were smokers, if they usually felt tired, if they followed a balanced diet, if they were physically active, or if they slept well.

The study was randomized, placebo-controlled, and double-blind. A laboratory member not directly involved in the present study conducted and completed the randomization of the participants and blinding of the study. First, treatments were coded as a placebo or supplement by flipping a coin. Then, placebo and supplement compounds were portioned in capsules with a similar appearance and consecutively numbered for each participant according to randomization. Each participant was assigned an order number and received the corresponding capsules. Both the participants and the investigators were blinded to the treatment order. Participants of the supplement group took two capsules daily for two months composed, for each capsule, of AM3-P (20%) (150 mg), spermidine (0.6 mg), hesperidin (50 mg), 2-hydrate calcium phosphate (299.07 mg), ZN sulfate (8.33 mg), and talcum powder (25 mg). In parallel, participants in the placebo group took two capsules, each composed of 2-hydrate calcium phosphate (500 mg) and talcum powder (25 mg), daily for two months.

Blood samples were obtained before the initiation of supplementation and after two months of treatment following the principles outlined in the Declaration of Helsinki. Specifically, 12 mL of peripheral blood was collected through venipuncture between 9:00 and 10:00 AM, using citrate-containing tubes (BD Vacutainer Systems), to control for circadian variations in immune parameters. All participants adhered to this schedule.

### 2.2. Analysis of Immune Function Parameters

#### 2.2.1. Isolation of Neutrophils and Lymphocytes

To assess neutrophil and lymphocyte chemotactic ability, neutrophil phagocytic activity, natural killer (NK) cell antitumoral function, and lymphoproliferative responses under both basal and stimulated conditions, neutrophils and lymphocytes were isolated from blood samples by following a previously established protocol [3]. Neutrophils and lymphocytes were isolated through density gradient centrifugation, utilizing Ficoll solutions with densities of 1.119 g/mL and 1.077 g/mL, respectively (Hystopaque, Sigma-Aldrich, St. Louis, MO, USA). The collected cells, exhibiting a viability of 95% as assessed by trypan blue exclusion, were subsequently adjusted to a concentration of 10^6^ neutrophils or lymphocytes per mL in an RPMI 1640 medium (Sigma-Aldrich).

#### 2.2.2. Chemotaxis

The chemotactic capacity of neutrophils and lymphocytes was evaluated by using a modified Boyden chamber technique, as previously described [3]. This approach assesses the ability of immune cells to migrate toward chemoattractants that mimic an infection site. Cell suspensions were introduced into the upper chamber, while f-met-leu-phe (Sigma-Aldrich) was placed in the lower chamber as the chemoattractant. Following a 3 h incubation period, filters were fixed and stained with Giemsa solution (Sigma-Aldrich). The chemotaxis index (C.I.) was calculated by counting the number of neutrophils or lymphocytes on one-third of the filter’s lower surface under optical microscopy at 100× magnification.

#### 2.2.3. Phagocytosis

For this analysis, a modified version of the technique originally described by De la Fuente was employed [3]. Neutrophil suspensions (200 μL) were incubated for 30 min in migration inhibition factor (MIF) plates, which promote cell adhesion, allowing neutrophils to form a monolayer at the well base. The adherent cells were washed with Hank’s solution at 37 °C, after which 20 μL of latex beads (1.09 μm, 1% suspension in PBS, Sigma-Aldrich) was added. Following a 30 min incubation, the samples were fixed in 50% methanol and stained with Giemsa solution (Sigma-Aldrich). Phagocytic activity was quantified by determining the phagocytic index, the number of particles ingested per 100 neutrophils, the phagocytic efficiency, and the percentage of neutrophils that had ingested at least one particle, using optical microscopy at 100× magnification.

#### 2.2.4. Natural Killer Antitumoral Activity

Natural killer (NK) cell activity was assessed by using an enzymatic colorimetric assay kit (Cytotox 96™; Promega, Madison, WI, USA) that quantifies lactate dehydrogenase (LDH) release, indicative of target cell cytolysis through the reduction of tetrazolium salts. A 100 µL aliquot of the NK cell suspension was combined with human K-562 lymphoma target cells in 96-well U-bottom plates at an effector-to-target cell ratio of 10:1. Following a 4 h incubation, LDH release was measured by adding the substrate for the enzyme reaction, and absorbance was recorded at 490 nm. NK cell antitumoral function was then calculated by using the following formula:

$$Lysis\;\%=\frac{Problem\;lysis-Effector\;cells\;spontaneous\;lysis-Tumor\;cells\;spontaneous\;lysis}{Tumor\;cells\;total\;lysis-Tumor\;cells\;spontaneous\;lysis}\times 100$$

As previously established, the results were quantified as the percentage of lysed tumor cells (% lysis) [3].

#### 2.2.5. Lymphoproliferation

Lymphocyte suspensions, adjusted to a concentration of 10^6^ cells/mL in complete RPMI medium (supplemented with 1 mg/mL gentamicin and 10% heat-inactivated fetal bovine serum (Gibco, Waltham, MA, USA), incubated at 56 °C for 30 min), were dispensed in 200 µL aliquots per well in 96-well culture plates. For basal conditions, 20 µL of RPMI medium was added to each well, while 20 µL of either phytohemagglutinin (PHA) or lipopolysaccharide (LPS) (1 µg/mL) was added to stimulate mitogenic responses. After 48 h of incubation, 100 µL from each well was collected for cytokine analysis. The removed volume was replenished with fresh medium, and tritiated thymidine (0.5 µCi/well) was added, followed by an additional 24 h incubation. The cells were then fixed onto filters by using an automated system, and the incorporation of tritiated thymidine was measured via a beta counter. Results were reported as counts per minute (c.p.m.) for basal and stimulated conditions [3].

### 2.3. Evaluation of Redox Parameters

To assess redox parameters, including glutathione reductase and peroxidase activities, concentrations of oxidized and reduced glutathione, thiobarbituric acid-reactive substances, whole-blood cells (comprising erythrocytes and leukocytes), plasma, and erythrocytes were used. Erythrocytes were isolated through Ficoll gradient centrifugation, initially performed to separate lymphocytes and neutrophils. Whole-blood cells and plasma were obtained by centrifuging blood samples at 1300× *g* for 20 min, after which the plasma and cellular fractions were carefully separated. The resulting cell pellets were re-suspended in RPMI+ medium. All samples were stored at −80 °C until further analysis.

#### 2.3.1. Glutathione Reductase Activity

The samples were re-suspended in an oxygen-free phosphate buffer (pH 7.4, 50 mM, with 6.3 nM EDTA), and then subjected to sonication followed by centrifugation. As outlined in prior protocols, the supernatants were used in the reaction, along with 80 mM GSSG as the substrate [59]. The oxidation of NADPH was monitored at 340 nm over 4 min. Results were reported as milliunits (mU) of glutathione reductase (GR) activity per milligram of protein.

#### 2.3.2. Glutathione Peroxidase Activity

The samples were re-suspended in an oxygen-free phosphate buffer (pH 7.4, 50 mM), followed by sonication and subsequent centrifugation. As previously detailed, the supernatants were collected and used in the enzymatic assay, with cumene hydroperoxide serving as the substrate [59]. NADPH oxidation was monitored at 340 nm over 5 min. The results were quantified as milliunits (mU) of glutathione peroxidase (GPx) activity per milligram of protein.

#### 2.3.3. Concentrations of Oxidized Glutathione (GSSG) and Reduced Glutathione (GSH)

The samples were re-suspended in phosphate buffer (pH 8, 50 mM; EDTA, 0.1 M) and then subjected to sonication and centrifugation. The supernatants were used to quantify both oxidized (GSSG) and reduced (GSH) glutathione based on their reactivity with o-phthalaldehyde at pH 12 and pH 8, respectively, which results in the formation of a fluorescent compound measurable at 420 nm, as described previously [59]. The results were expressed as nmol of GSSG and GSH per milligram of protein. Additionally, the GSSG/GSH ratio was calculated.

#### 2.3.4. Concentration of Thiobarbituric Acid-Reactive Substances (TBARs)

TBARs (thiobarbituric acid-reactive substances) were quantified by using a commercial Lipid Peroxidation Assay Kit (Biovision, San Francisco, CA, USA). The samples were re-suspended in a lysis buffer containing 0.1 mM butylated hydroxytoluene (BHT), sonicated, and centrifuged. The resulting supernatants were combined with thiobarbituric acid (TBA) and incubated in a water bath at 95 °C for 60 min. Following incubation, the samples were centrifuged, and the supernatants were collected. Absorbance was measured at 532 nm. The results were expressed as nanomoles of TBARs per milligram of protein.

#### 2.3.5. Protein Quantification

Protein concentration was determined in the same supernatants used for the analysis of various redox parameters. Protein quantification was performed by using the bicinchoninic acid (BCA) method, using a BCA kit. This method is based on the reduction of Cu^2+^ to Cu^+^ ions, which subsequently bind to BCA, forming a colored complex that absorbs light at 562 nm. The results were expressed as milligrams of protein per milliliter.

### 2.4. Biological Age Determination

The Immunity Clock model [4] was used to determine the biological age of each participant. The calculation for ImmunolAge is expressed by the following formula: ImmunolAge = 93.943 − 0.230 × natural killer activity − 0.001 × lymphoproliferative response to PHA − 0.022 × neutrophil chemotaxis − 0.020 × phagocytic index − 0.019 × lymphocyte chemotaxis.

### 2.5. Cytokine Measurement

Cytokine levels were measured in plasma and lymphocyte culture samples under basal conditions. The concentrations of TNF-α, IL-1β, IL-6, IL-10, and IL-2 were simultaneously quantified by using multiplex luminometry, employing a Milliplex^®^ MAP Human High Sensitivity T Cell Magnetic Bead Panel (HSTCMAG-28SK, Millipore, Burlington, MA, USA). The results were reported as picograms per milliliter (pg/mL).

### 2.6. Statistical Analysis

Statistical analyses were conducted by using GraphPad Prism 10.1.1 software. The normality of the data distributions was assessed by using the Kolmogorov–Smirnov test, while the homogeneity of variances was evaluated through Levene’s test. We compared demographics, health measures, perceived stress, anxiety, or resilience at the baseline by using the chi-squared test for categorical data and *t*-tests for continuous measures. Comparisons between initial and post-treatment conditions were conducted by using the dependent-samples *t*-test based on the normality of the data distribution. A *p*-value of less than 0.05 was considered statistically significant.

## 3. Results

**Figure 1:**
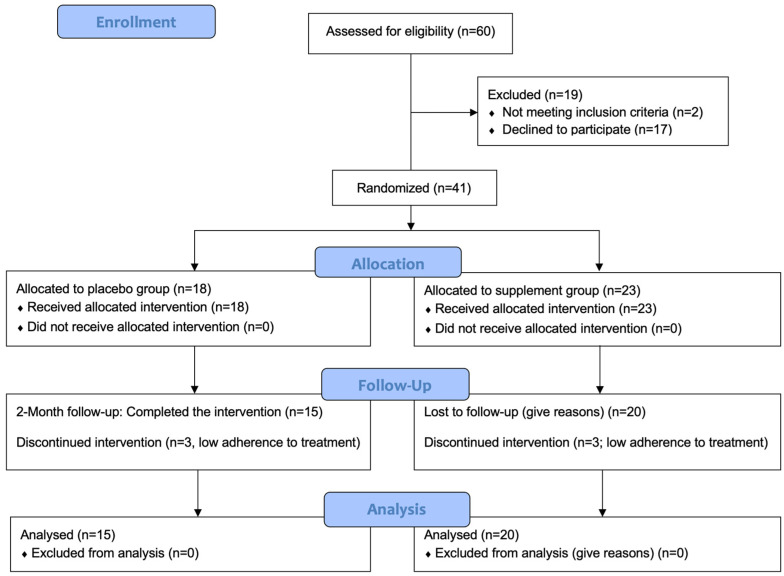
CONSORT flow diagram. CONSORT: consolidated standards of reporting trials.

We assessed 60 individuals for eligibility. Of these, 19 were excluded. We thus randomized 41 individuals in the placebo (N = 18) and supplement (N = 23) groups. Recruitment commenced in February 2022, and the final follow-up assessment was conducted in July 2022. At the time of completion of the study, six participants were excluded due to low adherence to the treatment. Therefore, there were 15 participants in the placebo group and 20 participants in the supplement group. All were included in each analysis. Figure 1 presents a CONSORT flow diagram. 

Baseline characteristics of enrolled participants who completed the study are displayed in Table 1. The placebo and supplement groups did not differ in terms of demographic, health measure, perceived stress, anxiety, or resilience data at the baseline (Table 1).

**Figure 2:**
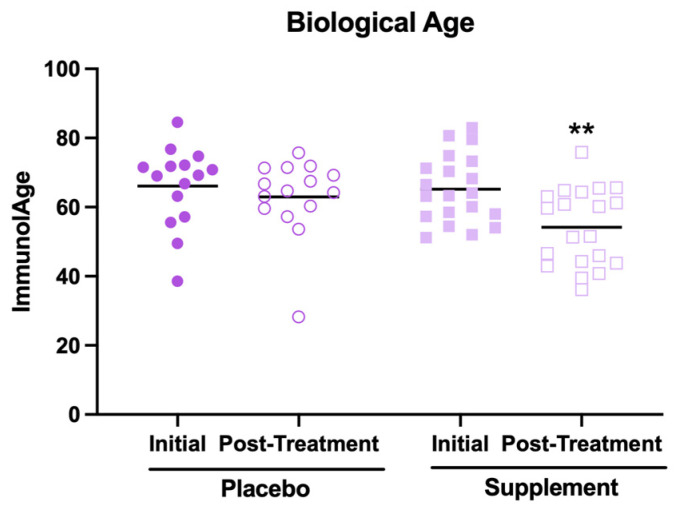
Biological age estimated through the Immunity Clock model in participants of the placebo and supplement groups before and after the treatment. ** *p* < 0.01 compared to the initial condition.

The results related to the effects of supplement intake on biological age are shown in Figure 2. First, it is worth mentioning that, in their initial condition, the participants showed a mean biological age higher than the mean of their chronological ages (66 ± 10 years versus 46 ± 8 years, respectively). This could be because most participants report at the baseline a medium perceived stress level and moderate anxiety, which are factors that have been associated with an accelerated rate of aging [4]. However, it can be observed that, after the supplementation, the participants decreased their biological age (Figure 2, *p* < 0.01) compared to the initial condition. In contrast, no effect was observed in the placebo group. However, despite this decrease in biological age, it was still higher than the average of their chronological ages (57 ± 11 years versus 46 ± 8 years, respectively).

**Figure 3:**
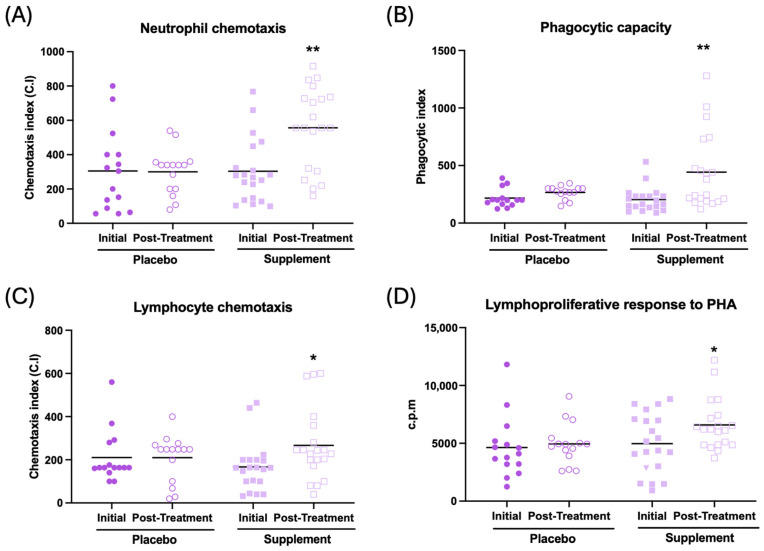
Immune function in peripheral blood leukocytes of participants in the placebo and supplement groups before and after the treatment. (**A**) Neutrophil chemotaxis (chemotaxis index). (**B**) Phagocytic capacity of neutrophils (phagocytic index). (**C**) Lymphocyte chemotaxis (chemotaxis index). (**D**) Lymphoproliferation in response to PHA (c.p.m). * *p* < 0.05, ** *p* < 0.01 compared to the initial condition. PHA: phytohemagglutinin; c.p.m: counts per minute.

The effects observed on immune function are illustrated in Figure 3 and Table 2. In general, an improvement in immune function can be observed after supplementation. Thus, an increase in neutrophil and lymphocyte chemotaxis (Figure 3A, *p* < 0.01; Figure 3C, *p* < 0.05, respectively), an increase in the phagocytic index and efficacy (Figure 3B, *p* < 0.01; Table 2, *p* < 0.01, respectively), and increased lymphoproliferation in response to phytohemagglutinin (PHA) and lipopolysaccharide (Figure 3D, *p* < 0.05; Table 2, *p* < 0.05, respectively) can be observed in the supplemented group compared to their initial conditions. There was no effect in NK activity after supplementation (Table 2). No differences were observed between the baseline and post-treatment conditions in the placebo group.

**Figure 4:**
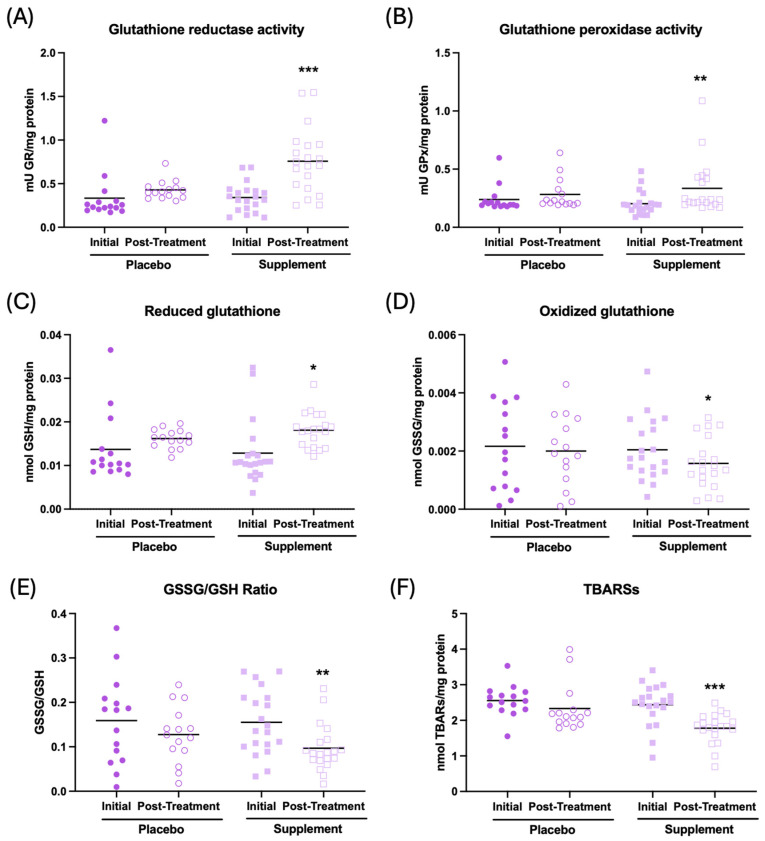
Redox parameters assessed in whole-blood cells of participants in the placebo and supplement groups before and after the treatment. (**A**) Glutathione reductase activity (mU GR/mg protein). (**B**) Glutathione peroxidase activity (mU GPx/mg protein). (**C**) Concentration of reduced glutathione (nmol GSH/mg protein). (**D**) Concentration of oxidized glutathione (nmol GSSG/mg protein). (**E**) GSSG/GSH ratio. (**F**) Thiobarbituric acid-reactive substance concentration (nmol TBARs/mg protein). * *p* < 0.05, ** *p* < 0.01, and *** *p* < 0.001 compared to the initial condition. GR: glutathione reductase activity; GPx: glutathione peroxidase activity; GSSG: oxidized glutathione; GSH: reduced glutathione; and TBARs: thiobarbituric acid-reactive substances.

The results of the effect of supplementation on the redox parameters are shown in Figure 4. It can be observed that, after supplementation, the participants show an increase in the antioxidant activities of glutathione reductase and peroxidase (Figure 4A, *p* < 0.001; Figure 4B, *p* < 0.01, respectively), an increase in the concentration of reduced glutathione (GSH) c (Figure 4C, *p* < 0.05), a decrease in the concentration of oxidized glutathione (GSSG) (Figure 4D, *p* < 0.05), and a lower GSSG/GSH ratio (Figure 4E, *p* < 0.01), as well as a decrease in oxidative lipid damage (TBARs) (Figure 4F, *p* < 0.001) in the whole-blood cell sample with respect to the initial condition. There were no differences in the placebo group after treatment. Similar results can be observed in plasma and erythrocyte samples, as shown in Supplementary Table S1.

**Figure 5:**
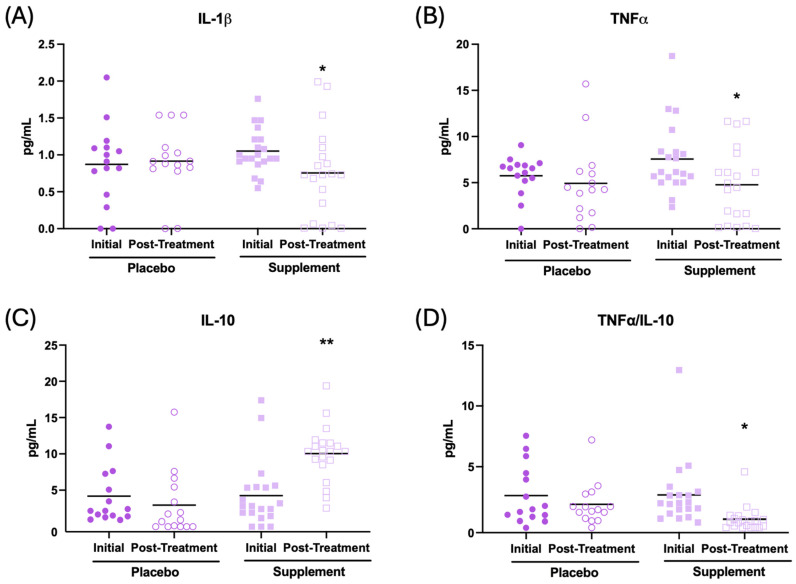
Cytokine concentrations (pg/mL) in the plasma of participants in the placebo and supplement groups before and after the treatment. (**A**) IL-1β concentration. (**B**) TNF-α concentration. (**C**) IL-10 concentration. (**D**) TNF-α/IL-10 ratio. * *p* < 0.05, ** *p* < 0.01 compared to the initial condition.

In addition, the effect of the supplement on the inflammatory state in the plasma of the patients was evaluated. The results are presented in Figure 5 and Table 2. After supplementation, participants had their IL-1β concentration (Figure 5A, *p* < 0.05), TNF-α (Figure 5B, *p* < 0.05), and IL-2 (Table 2, *p* < 0.01) decrease in relation to the initial condition. In addition, an increase in IL-10 (Figure 5C, *p* < 0.01) and IL-6 (Table 2, *p* < 0.001), along with a lower TNF-α/IL-10 ratio (Figure 5D, *p* < 0.05), were observed after supplementation with respect to the initial condition. No significant changes were observed in the placebo group after treatment.

Finally, inflammatory parameters were also evaluated in the supernatants of mononuclear cell cultures under basal conditions, as shown in Table 2. It can be observed that, after supplementation, participants decreased their lymphoproliferation in basal conditions (Table 2, *p* < 0.05), as well as the concentration of IL-1β (Table 2, *p* < 0.001) and the TNF-α/IL-10 ratio (Table 2, *p* < 0.01) with respect to the initial condition. An increase in IL-6 concentration (Table 2, *p* < 0.001) was also observed. No evidence of a placebo effect was detected.

## 4. Discussion

This study is the first to demonstrate the effect that the daily consumption for two months of a supplement with AM3, spermidine, and hesperidin can have on immunity, oxidative state, inflammatory profile, and rate of aging.

The results of this study show that, after taking the supplement, certain immune functions are improved. Specifically, it has been observed that, after this supplementation, there is an improvement in the chemotaxis of neutrophils and lymphocytes, in the phagocytic capacity of neutrophils, and in the lymphoproliferative response to mitogens. These results agree with those obtained in other studies, since there is evidence that oral supplementation with AM3 can restore immune impairment (or immunosenescence), enhancing the functionality of NK cells and monocytes [23]. Moreover, hesperidin shows antioxidant and anti-inflammatory properties, downregulating the production of pro-inflammatory cytokines such as IL-8 and TNF-α [51], which would also explain the improvement of these immune functions as oxidative and inflammatory stress are fundamental drivers of immunosenescence [1]. Similarly, spermidine also has anti-inflammatory effects and induces autophagy, thus improving immune functionality in old age [60]. However, although supplementation would be expected to also potentiate NK activity, no differences in this parameter were observed. This could be because spermidine shows an inhibitory effect on NK activity by transforming into spermine, which is finally transformed into active fractions that suppress NK activity [61,62].

Although the supplement does not affect NK activity, the overall enhancement of immune function is also reflected in the rejuvenation of the biological age of the participants since the functions of neutrophil and lymphocyte chemotaxis, neutrophil phagocytosis, and proliferation in response to PHA are included in the Immunity Clock mathematical model, which was used to calculate the biological age [4]. This is of great importance, since this is the first study that shows that this supplement could help to reduce the biological age, leading an individual to have a better aging process and achieve healthy longevity [1,2,3,4]. Moreover, this has certain clinical implications since chronological age does not always coincide with biological age, which indicates how fast we are aging. In fact, people usually show a higher biological age than their chronological age due to different situations in their daily lives, especially those related to stress and anxiety [1,2,3,4]. Therefore, finding supplements, such as the one evaluated in the present study, that allow for the control of the rate of aging is of great interest in favor of the healthy aging of the population. Nevertheless, this is not the only food supplement that can reduce biological age by improving immune function and redox as well as inflammatory profiles. It has been observed that other antioxidant supplements or different probiotic blends act similarly [4,63].

In this line, it has been suggested that chronic oxidative stress, coupled with inflammatory stress, constitutes the primary cause of aging, as these factors contribute to cellular damage, particularly in homeostatic systems such as the nervous, endocrine, and immune systems, which explains the increased risk of morbidity and mortality that occurs with aging. Furthermore, the immune system is considered a crucial modulator of the interplay between oxidation and inflammation, and, consequently, the rate at which aging occurs in individuals [1,2]. Thus, the functional state of immunity has an impact on “oxi-inflammaging” and the achieved life span [1,2].

Among the parameters used to assess the redox state, those related to the glutathione cycle have been identified as potential biomarkers for the rate of aging and longevity [64]. Specifically, enzymatic activities of glutathione reductase (GR) and peroxidase (GPx), along with the concentrations of oxidized (GSSG) and reduced (GSH) glutathione in human peripheral blood leukocytes, have been shown to correlate with biological age determined by the Immunity Clock [59].

The findings from the current study indicate that after taking a nutritional supplement rich in AM3, hesperidin, and spermidine daily for 2 months, there is a decrease in oxidative stress since antioxidant defenses, such as GPx and GR activities, increase and oxidants decrease, as seen in the concentration of GSSG, which implies a decrease in the GSSG/GSH ratio, a reliable indicator of the oxidative state [65] and oxidative damage to lipids (TBARs). This decrease in the oxidative stress of the participants after taking the supplement could translate, as mentioned above, into an improvement of the homeostatic systems, reducing the risk of morbidity and mortality that appears with aging [1,2]. These results seem to be mainly mediated by spermidine and hesperidin, as the antioxidant capacity of these compounds has been previously described [27,28,29,30,31,32,33,34,35,51,53,54,55,56,60]. In contrast, the antioxidant properties of AM3 have been less studied.

The general inflammatory state of the organism can be reflected in the pro-inflammatory and anti-inflammatory cytokines circulating in the blood. These cytokines do not necessarily originate from immune cells but also from multiple cells of different organs and systems. In this study, it has been found that ingestion of the supplement produces a decrease in the plasma of typically pro-inflammatory cytokines, such as TNF-α and IL-1β [66], and an increase in the anti-inflammatory IL-10, so that the TNF-α/IL-10 ratio, an excellent indicator of the inflammatory state of an organism [67], decreases. The increase observed in IL-6, considered pro-inflammatory [68], is a curious result. However, it should be considered that it has been demonstrated that this cytokine, released by senescent cells, may have an important role in cellular reprogramming, a fundamental factor in improving cellular repair and aging [69]. Similarly, when the inflammatory state is analyzed in mononuclear cell cultures in basal conditions, a decrease in basal lymphoproliferation is observed, an indicator of the decrease in sterile inflammation generated by the immune cells [67]. Thus, a decrease in IL-1β and the TNF-α/IL-10 ratio is observed, showing, as in plasma, that this supplement has anti-inflammatory properties. An increase in IL-6 is also observed here, which could be related to cell reprogramming processes [69]. These anti-inflammatory properties shown by the supplement are not surprising, as it has previously been described that both AM3, spermidine, and hesperidin exhibit this property [27,28,29,30,31,32,33,34,35,51,53,54,55,56,60]. However, this anti-inflammatory property of the supplement is of great importance, as it allows for the control of the inflammatory stress that occurs during the aging process, helping to slow it down [1,2].

Altogether, considering that the onset of oxidative and inflammatory stress, primarily driven by the immune system, underlies the process of aging [1], the supplement’s effect on enhancing immune function, modulating oxidative and inflammatory states, and slowing the rate of aging could translate into an increase in the longevity of the individual [64]. Indeed, prior research has demonstrated that some of the components of this nutritional supplement can affect longevity. Thus, hesperidin and spermidine have been observed to increase life span, with the latter performing this via autophagy [60,70,71,72]. However, it is not known whether this supplement, consisting of AM3, spermidine, and hesperidin, could increase longevity.

Therefore, different limitations could be described in the present study. Firstly, the small sample size of the study and the treatment time should be mentioned. In future studies, it would be appropriate to work with a larger sample size, which would allow us to obtain greater statistical power and study potential inter-individual differences in response to the supplement. It would also be interesting to see if the effects are maintained in the long term after supplementation and, if not, to study other concentrations or other intervention times. In addition, in the present study, the participants were advised to continue with their usual diet throughout the treatment, and the daily intake of antioxidants through the diet was not taken into account, which would be something to consider in the future. Moreover, it would be convenient to carry out studies in experimental animals to corroborate whether the positive effects observed in the present study are produced and, if so, if the animals supplemented achieve greater longevity. Moreover, given that experimental animals do not show a placebo effect, carrying out this study in animals could corroborate that the supplement does not have this effect, as observed in the present work.

## 5. Conclusions

From the results obtained, it can be concluded that volunteers who took two capsules of a daily supplement of AM3 (150 mg), spermidine (0.6 mg), and hesperidin (50 mg) for 2 months decreased their biological age, with an average rejuvenation of 11 years. Moreover, they stimulated relevant immune functions, such as chemotaxis, phagocytosis, and lymphoproliferative responses to the mitogen PHA. Furthermore, they also improved their redox state and positively modulated the general inflammatory state of organisms. Therefore, the ingestion of this supplement could be proposed as a strategy to maintain better health and slow down the rate of aging, which would allow them to achieve greater longevity.

## Figures and Tables

**Table 1:** Baseline characteristics of individuals in the placebo (N = 15) and supplement (N = 20) groups.

	Placebo (N = 15)	Supplement (N = 20)
**Demographic Variables**		
Gender N (% females)	8 (53.3%)	10 (50%)
Age M (SD)	46.4 (6.2)	47.1 (6.9)
**Health Measures**		
Current smoker N (%)	0 (0%)	4 (20%)
Currently tired N (%)	4 (26.6%)	2 (10%)
Balanced diet N (%)	13 (86.6%)	17 (85%)
Physical activity N (%)	14 (93.3%)	17 (85%)
Sleep well N (%)	12 (80%)	15 (75%)
**Perceived Stress**		
Low N (%)	2 (13.3%)	8 (40%)
Medium N (%)	13 (86.7%)	12 (60%)
**Anxiety**		
Low N (%)	1 (6.6%)	1 (5%)
Moderate N (%)	14 (93.4%)	19 (95%)
**Resilience**		
High N (%)	13 (86.7%)	20 (100%)
Moderate N (%)	2 (13.3%)	0 (0%)

**Table 2:** Immune function parameters in peripheral blood leukocytes and inflammation parameters in monocyte cultures at basal conditions and in the plasma of participants in the placebo and supplement groups before and after the treatment.

	Placebo		Supplement	
	Initial	Post-Treatment	Initial	Post-Treatment
**Immune Function Parameters**				
Phagocytic efficacy	65 ± 6	69 ± 7	62 ± 10	75 ± 15 **
Natural Killer activity (% Lysis of tumoral cells)	51.8 ± 26.2	50.5 ± 22.1	58.8 ± 26	52.4 ± 23.1
Proliferative response to LPS (c.p.m)	4637 ± 2651	4952 ± 1783	4973 ± 2556	6594 ± 2199 *
**Inflammation Parameters in Plasma**				
IL-6 concentration (pg/mL)	2.6 ± 2.2	3.7 ± 2.8	4.8 ± 3.8	10.4 ± 6.7 ***
IL-2 concentration (pg/mL)	2.4 ± 2	1.9 ± 1.4	2.3 ± 1.3	0.9 ± 0.6 **
**Inflammation Parameters in Monocyte Cultures**				
Basal proliferative response (c.p.m)	607 ± 243	577 ± 232	686 ± 131	580 ± 216 *
TNFα concentration (pg/mL)	340 ± 183	494 ± 143	350 ± 177	386 ± 132
IL-1β concentration (pg/mL)	759 ± 571	994 ± 180	749 ± 593	573 ± 202 ***
IL-6 concentration (pg/mL)	1109 ± 337	1283 ± 266	930 ± 418	1311 ± 251 ***
TNFα/IL-10 ratio (pg/mL)	0.68 ± 0.62	0.53 ± 0.46	0.64 ± 0.54	0.32 ± 0.26 **

Values are presented as the mean ± standard deviation. * *p* < 0.05, ** *p* < 0.01, and *** *p* < 0.001, indicating statistical significance compared to the initial condition. LPS: lipopolysaccharide.

## Data Availability

Data will be available upon request to Judith Félix (jufelix@ucm.es).

## Supplementary Materials

The following supporting information can be downloaded at: https://www.mdpi.com/article/10.3390/antiox13111391/s1, Table S1: Redox parameters in the erythrocytes and plasma of participants of the placebo and supplement groups before and after the treatment. CONSORT checklist.

## References

[1] De la Fuente M., Miquel J. (2009). An update of the oxidation-inflammation theory of aging: The involvement of the immune system in oxi-inflamm-aging. Curr. Pharm. Des..

[2] Martínez de Toda I., Ceprián N., Díaz-Del Cerro E., De la Fuente M. (2021). The Role of Immune Cells in Oxi-Inflamm-Aging. Cells.

[3] Martínez de Toda I., Maté I., Vida C., Cruces J., De la Fuente M. (2016). Immune function parameters as markers of biological age and predictors of longevity. Aging.

[4] Martínez de Toda I., Vida C., Díaz-Del Cerro E., De la Fuente M. (2021). The Immunity Clock. J. Gerontol. A Biol. Sci. Med. Sci..

[5] De la Fuente M., Hernanz A., Guayerbas N., Victor V.M., Arnalich F. (2008). Vitamin E ingestion improves several immune functions in elderly men and women. Free Radic. Res..

[6] Baeza I., De Castro N.M., Arranz L., De la Fuente M. (2010). Soybean and green tea polyphenols improve immune function and redox status in very old ovariectomized mice. Rejuvenation Res..

[7] Rojo J.M., Rejas M.T., Ojeda G., Portolés P., Barasoain I. (1986). Enhancement of lymphocyte proliferation, interleukin-2 production and NK activity by inmunoferon (AM-3), a fungal immunomodulator: Variations in normal and immunosuppressed mice. Int. J. Immunopharmacol..

[8] Moya P., Baixeras E., Barasoain I., Rojo J.M., Ronda E., Alonso M.L., Portolés A. (1987). Immunoferon (AM3) enhances the activities of early-type interferon inducers and natural killer cells. Immunopharmacol. Immunotoxicol..

[9] Sánchez Palacios A., García Marrero J.A., Schamann F. (1992). Immunologic clinical evaluation of a biological response modifier, AM3, in the treatment of childhood infectious respiratory pathology. Allergol. Immunopathol..

[10] Sánchez L., Peña E., Civantos A., Sada G., Alvarez M.M., Chirigos M.A., Villarrubia V.G. (1995). AM3, an adjuvant to hepatitis B revaccination in non-responder healthy persons. J. Hepatol..

[11] Pérez-García R., Pérez-García A., Verbeelen D., Bernstein E.D., Villarrubia V.G., Alvarez-Mon M. (2002). AM3 (Inmunoferón) as an adjuvant to hepatitis B vaccination in hemodialysis patients. Kidney Int..

[12] Alvarez-Mon M., Miravitlles M., Morera J., Callol L., Alvarez-Sala J.L. (2005). Treatment with the immunomodulator AM3 improves the health-related quality of life of patients with COPD. Chest.

[13] Serrano-Gómez D., Martínez-Nuñez R.T., Sierra-Filardi E., Izquierdo N., Colmenares M., Pla J., Rivas L., Martinez-Picado J., Jimenez-Barbero J., Alonso-Lebrero J.L. (2007). AM3 modulates dendritic cell pathogen recognition capabilities by targeting DC-SIGN. Antimicrob. Agents Chemother..

[14] Martín-Vilchez S., Molina-Jiménez F., Alonso-Lebrero J.L., Sanz-Cameno P., Rodríguez-Muñoz Y., Benedicto I., Roda-Navarro P., Trapero M., Aragoneses-Fenoll L., González S. (2008). AM3, a natural glycoconjugate, induces the functional maturation of human dendritic cells. Br. J. Pharmacol..

[15] Albillos A., Nieto M., Ubeda M., Muñoz L., Fraile B., Reyes E., Lledó L., Blanco I., Pastor O., Salas C. (2010). The biological response modifier AM3 attenuates the inflammatory cell response and hepatic fibrosis in rats with biliary cirrhosis. Gut.

[16] Yuan C.L., Lin S.W., Cheng M.H. (2016). Inhibition of Molecular Signaling in Huh-7 Cells by AM3: A Novel Chemotherapeutic Agent for Hepatocellular Carcinoma. Med. Chem..

[17] Geckin B., Kilic G., Debisarun P.A., Föhse K., Rodríguez-Luna A., Fernández-González P., Sánchez A.L., Domínguez-Andrés J. (2023). The fungal-derived compound AM3 modulates pro-inflammatory cytokine production and skews the differentiation of human monocytes. Front. Immunol..

[18] Jimenez-Gómez N., López-Suárez A., Haro S., Fernández-González P., Monserrat J., Eraña-Tomás I., Cuevas-Santos J., Rodríguez-Luna A., Ortega M.A., Gómez-Sánchez M.J. (2024). Immunomodulation with AM3 and antioxidants creates an adequate framework for skin repair and decreases the monocyte proinflammatory stage in smoker women. Biomed. Pharmacother..

[19] Prieto A., Reyes E., Bernstein E.D., Martinez B., Monserrat J., Izquierdo J.L., Callol L., de L.P., Alvarez-Sala R., Alvarez-Sala J.L. (2001). Defective natural killer and phagocytic activities in chronic obstructive pulmonary disease are restored by glycophosphopeptical (inmunoferón). Am. J. Respir. Crit. Care Med..

[20] Reyes E., Prieto A., de la Hera A., de Lucas P., Alvarez-Sala R., Alvarez-Sala J.L., Alvarez-Mon M. (2006). Treatment with AM3 restores defective T-cell function in COPD patients. Chest.

[21] Córdova A., Sureda A., Pons A., Alvarez-Mon M. (2015). Modulation of TNF-α, TNF-α receptors and IL-6 after treatment with AM3 in professional cyclists. J. Sports Med. Phys. Fit..

[22] Fernández-Lázaro D., Fernandez-Lazaro C.I., Mielgo-Ayuso J., Adams D.P., García Hernández J.L., González-Bernal J., González-Gross M. (2021). Glycophosphopeptical AM3 Food Supplement: A Potential Adjuvant in the Treatment and Vaccination of SARS-CoV-2. Front. Immunol..

[23] Villarrubia V.G., Moreno Koch M.C., Calvo C., González S., Alvarez-Mon M. (1997). The immunosenescent phenotype in mice and humans can be defined by alterations in the natural immunity reversal by immunomodulation with oral AM3. Immunopharmacol. Immunotoxicol..

[24] Majano P., Alonso-Lebrero J.L., Janczyk A., Martín-Vichez S., Molina-Jiménez F., Brieva A., Pivel J.P., González S., López-Cabrera M., Moreno-Otero R. (2005). AM3 inhibits LPS-induced iNOS expression in mice. Int. Immunopharmacol..

[25] Yu L., Pan J., Guo M., Duan H., Zhang H., Narbad A., Zhai Q., Tian F., Chen W. (2023). Gut microbiota and anti-aging: Focusing on spermidine. Crit. Rev. Food Sci. Nutr..

[26] Guarente L., Sinclair D.A., Kroemer G. (2024). Human trials exploring anti-aging medicines. Cell Metab..

[27] Larqué E., Sabater-Molina M., Zamora S. (2007). Biological significance of dietary polyamines. Nutrition.

[28] Ramani D., De Bandt J.P., Cynober L. (2014). Aliphatic polyamines in physiology and diseases. Clin. Nutr..

[29] Hesterberg R.S., Cleveland J.L., Epling-Burnette P.K. (2018). Role of Polyamines in Immune Cell Functions. Med. Sci..

[30] Handa A.K., Fatima T., Mattoo A.K. (2018). Polyamines: Bio-Molecules with Diverse Functions in Plant and Human Health and Disease. Front. Chem..

[31] Muñoz-Esparza N.C., Latorre-Moratalla M.L., Comas-Basté O., Toro-Funes N., Veciana-Nogués M.T., Vidal-Carou M.C. (2019). Polyamines in Food. Front. Nutr..

[32] Ramos-Molina B., Queipo-Ortuño M.I., Lambertos A., Tinahones F.J., Peñafiel R. (2019). Dietary and Gut Microbiota Polyamines in Obesity- and Age-Related Diseases. Front. Nutr..

[33] Nakanishi S., Cleveland J.L. (2021). Polyamine Homeostasis in Development and Disease. Med. Sci..

[34] Sagar N.A., Tarafdar S., Agarwal S., Tarafdar A., Sharma S. (2021). Polyamines: Functions, Metabolism, and Role in Human Disease Management. Med. Sci..

[35] Négrel S., Brunel J.M. (2021). Synthesis and Biological Activities of Naturally Functionalized Polyamines: An Overview. Curr. Med. Chem..

[36] Niechcial A., Schwarzfischer M., Wawrzyniak M., Atrott K., Laimbacher A., Morsy Y., Katkeviciute E., Häfliger J., Westermann P., Akdis C.A. (2023). Spermidine Ameliorates Colitis via Induction of Anti-Inflammatory Macrophages and Prevention of Intestinal Dysbiosis. J. Crohns Colitis.

[37] Ueno D., Ikeda K., Yamazaki E., Katayama A., Urata R., Matoba S. (2023). Spermidine improves angiogenic capacity of senescent endothelial cells, and enhances ischemia-induced neovascularization in aged mice. Sci. Rep..

[38] Hibino S., Eto S., Hangai S., Endo K., Ashitani S., Sugaya M., Osawa T., Soga T., Taniguchi T., Yanai H. (2023). Tumor cell-derived spermidine is an oncometabolite that suppresses TCR clustering for intratumoral CD8(+) T cell activation. Proc. Natl. Acad. Sci. USA.

[39] Zimmermann A., Hofer S.J., Madeo F. (2023). Molecular targets of spermidine: Implications for cancer suppression. Cell Stress.

[40] Đorđievski S., Vukašinović E.L., Čelić T.V., Pihler I., Kebert M., Kojić D., Purać J. (2023). Spermidine dietary supplementation and polyamines level in reference to survival and lifespan of honey bees. Sci. Rep..

[41] Coeli-Lacchini F.B., da Silva G., Belentani M., Alves J.S.F., Ushida T.R., Lunardelli G.T., Garcia C.B., Silva T.A., Lopes N.P., Leopoldino A.M. (2023). Spermidine Suppresses Oral Carcinogenesis through Autophagy Induction, DNA Damage Repair, and Oxidative Stress Reduction. Am. J. Pathol..

[42] Barreca D., Gattuso G., Bellocco E., Calderaro A., Trombetta D., Smeriglio A., Laganà G., Daglia M., Meneghini S., Nabavi S.M. (2017). Flavanones: Citrus phytochemical with health-promoting properties. Biofactors.

[43] Mirzaei A., Mirzaei A., Najjar Khalilabad S., Askari V.R., Baradaran Rahimi V. (2023). Promising influences of hesperidin and hesperetin against diabetes and its complications: A systematic review of molecular, cellular, and metabolic effects. Excli. J..

[44] Madureira M.B., Concato V.M., Cruz E.M.S., Bitencourt de Morais J.M., Inoue F.S.R., Concimo Santos N., Gonçalves M.D., Cremer de Souza M., Basso Scandolara T., Fontana Mezoni M. (2023). Naringenin and Hesperidin as Promising Alternatives for Prevention and Co-Adjuvant Therapy for Breast Cancer. Antioxidants.

[45] Li S., Hao L., Hu X., Li L. (2023). A systematic study on the treatment of hepatitis B-related hepatocellular carcinoma with drugs based on bioinformatics and key target reverse network pharmacology and experimental verification. Infect. Agents Cancer.

[46] Hosawi S. (2023). Current Update on Role of Hesperidin in Inflammatory Lung Diseases: Chemistry, Pharmacology, and Drug Delivery Approaches. Life.

[47] Kaviani F., Baratpour I., Ghasemi S. (2023). The Antidiabetic Mechanisms of Hesperidin: Hesperidin Nanocarriers as Promising Therapeutic Options for Diabetes. Curr. Mol. Med..

[48] Shylaja H., Viswanatha G.L., Sunil V., Hussain S.M., Farhana S.A. (2024). Effect of hesperidin on blood pressure and lipid profile: A systematic review and meta-analysis of randomized controlled trials. Phytother. Res..

[49] Ji Z., Deng W., Chen D., Liu Z., Shen Y., Dai J., Zhou H., Zhang M., Xu H., Dai B. (2024). Recent understanding of the mechanisms of the biological activities of hesperidin and hesperetin and their therapeutic effects on diseases. Heliyon.

[50] Bansal K., Singh V., Singh S., Mishra S. (2024). Neuroprotective Potential of Hesperidin as Therapeutic Agent in the Treatment of Brain Disorders: Preclinical Evidence-based Review. Curr. Mol. Med..

[51] Lee H.J., Im A.R., Kim S.M., Kang H.S., Lee J.D., Chae S. (2018). The flavonoid hesperidin exerts anti-photoaging effect by downregulating matrix metalloproteinase (MMP)-9 expression via mitogen activated protein kinase (MAPK)-dependent signaling pathways. BMC Complement. Altern. Med..

[52] Morshedzadeh N., Ramezani Ahmadi A., Behrouz V., Mir E. (2023). A narrative review on the role of hesperidin on metabolic parameters, liver enzymes, and inflammatory markers in nonalcoholic fatty liver disease. Food Sci. Nutr..

[53] Xiong H., Wang J., Ran Q., Lou G., Peng C., Gan Q., Hu J., Sun J., Yao R., Huang Q. (2019). Hesperidin: A Therapeutic Agent For Obesity. Drug Des. Dev. Ther..

[54] Tejada S., Pinya S., Martorell M., Capó X., Tur J.A., Pons A., Sureda A. (2018). Potential Anti-inflammatory Effects of Hesperidin from the Genus Citrus. Curr. Med. Chem..

[55] Hajialyani M., Hosein Farzaei M., Echeverría J., Nabavi S.M., Uriarte E., Sobarzo-Sánchez E. (2019). Hesperidin as a Neuroprotective Agent: A Review of Animal and Clinical Evidence. Molecules.

[56] Li C., Schluesener H. (2017). Health-promoting effects of the citrus flavanone hesperidin. Crit. Rev. Food Sci. Nutr..

[57] Estruel-Amades S., Massot-Cladera M., Pérez-Cano F.J., Franch À., Castell M., Camps-Bossacoma M. (2019). Hesperidin Effects on Gut Microbiota and Gut-Associated Lymphoid Tissue in Healthy Rats. Nutrients.

[58] Camps-Bossacoma M., Franch À., Pérez-Cano F.J., Castell M. (2017). Influence of Hesperidin on the Systemic and Intestinal Rat Immune Response. Nutrients.

[59] Diaz-Del Cerro E., Martinez de Toda I., Félix J., Baca A., De la Fuente M. (2023). Components of the Glutathione Cycle as Markers of Biological Age: An Approach to Clinical Application in Aging. Antioxidants.

[60] Zhang H., Simon A.K. (2020). Polyamines reverse immune senescence via the translational control of autophagy. Autophagy.

[61] Lian J., Liang Y., Zhang H., Lan M., Ye Z., Lin B., Qiu X., Zeng J. (2022). The role of polyamine metabolism in remodeling immune responses and blocking therapy within the tumor immune microenvironment. Front. Immunol..

[62] Zhang M., Wang H., Tracey K.J. (2000). Regulation of macrophage activation and inflammation by spermine: A new chapter in an old story. Crit. Care Med..

[63] Félix J., Baca A., Taboada L., Álvarez-Calatayud G., De la Fuente M. (2024). Consumption of a Probiotic Blend with Vitamin D Improves Immunity, Redox, and Inflammatory State, Decreasing the Rate of Aging—A Pilot Study. Biomolecules.

[64] Félix J., Martínez de Toda I., Díaz-Del Cerro E., Gil-Agudo F., De la Fuente M. (2024). The immunity and redox clocks in mice, markers of lifespan. Sci. Rep..

[65] Kanďár R. (2016). The ratio of oxidized and reduced forms of selected antioxidants as a possible marker of oxidative stress in humans. Biomed. Chromatogr..

[66] Tylutka A., Walas Ł., Zembron-Lacny A. (2024). Level of IL-6, TNF, and IL-1β and age-related diseases: A systematic review and meta-analysis. Front. Immunol..

[67] Martínez de Toda I., Vida C., De la Fuente M. (2017). An Appropriate Modulation of Lymphoproliferative Response and Cytokine Release as Possible Contributors to Longevity. Int. J. Mol. Sci..

[68] Al-Qahtani A.A., Alhamlan F.S., Al-Qahtani A.A. (2024). Pro-Inflammatory and Anti-Inflammatory Interleukins in Infectious Diseases: A Comprehensive Review. Trop. Med. Infect. Dis..

[69] Mosteiro L., Pantoja C., de Martino A., Serrano M. (2018). Senescence promotes in vivo reprogramming through p16(INK)(4a) and IL-6. Aging Cell.

[70] Eisenberg T., Abdellatif M., Schroeder S., Primessnig U., Stekovic S., Pendl T., Harger A., Schipke J., Zimmermann A., Schmidt A. (2016). Cardioprotection and lifespan extension by the natural polyamine spermidine. Nat. Med..

[71] Ge Y., Chen H., Wang J., Liu G., Cui S.W., Kang J., Jiang Y., Wang H. (2021). Naringenin prolongs lifespan and delays aging mediated by IIS and MAPK in Caenorhabditis elegans. Food Funct..

[72] Xu Q., Fu Q., Li Z., Liu H., Wang Y., Lin X., He R., Zhang X., Ju Z., Campisi J. (2021). The flavonoid procyanidin C1 has senotherapeutic activity and increases lifespan in mice. Nat. Metab..

