# Supplementary material for: Human Supplementation with AM3, Spermidine, and Hesperidin Enhances Immune Function, Decreases Biological Age, and Improves Oxidative–Inflammatory State: A Randomized Controlled Trial
Source: Antioxidants (Basel). 2024 Nov 14;13(11):1391. doi: 10.3390/antiox13111391 (PMC11591374; doi:10.3390/antiox13111391)
Supplement: Supplementary file 1 [file antioxidants-13-01391-s001.zip › Supplementary material.pdf]

Table S1. Redox parameters in erythrocytes and plasma of participants of the placebo and the supplement group before and after the treatment.

	Placebo		Supplement	
	**Initial**	**Post-Treatment**	**Initial**	**Post-Treatment**
**Erythrocytes**				
**Antioxidant compounds**				
Glutathione Reductase activity (mU GR/mg protein)	0.02±0.006	0.02±0.007	0.02±0.006	0.03±0.01*
Glutathione Peroxidase activity (mU GPx/mg protein)	0.9±0.2	1.1±0.4	0.9±0.3	2.3±1.9**
Reduced glutathione concentration (nmol GSH/mg protein)	0.01±0.006	0.01±0.003	0.01±0.007	0.02±0.005*
**Oxidant compounds**				
Oxidized glutathione concentration (nmol GSSG/mg protein)	0.004±0.003	0.005±0.003	0.004±0.001	0.004±0.004
TBARs concentration (nmol TBARs/mg protein)	1.9±0.5	1.6±0.9	1.9±0.4	0.7±0.3***
**GSSG/GSH Ratio**	0.4±0.1	0.5±0.4	0.4±0.2	0.2±0.1*
**Plasma**				
**Antioxidant compounds**				
Glutathione Reductase activity (mU GR/mg protein)	0.05±0.02	0.06±0.02	0.05±0.02	0.1±0.04**
Glutathione Peroxidase activity (mU GPx/mg protein)	0.03±0.01	0.03±0.01	0.02±0.01	0.05±0.02***
Reduced glutathione concentration (nmol GSH/mg protein)	0.003±0.001	0.004±0.002	0.004±0.002	0.01±0.006***
**Oxidant compounds**				
Oxidized glutathione concentration (nmol GSSG/mg protein)	0.008±0.006	0.007±0.003	0.008±0.007	0.004±0.003*
TBARs concentration (nmol TBARs/mg protein)	0.03±0.01	0.03±0.01	0.03±0.01	0.02±0.01*
**GSSG/GSH Ratio**	1.5±0.5	1.2±0.8	1.2±0.6	0.5±0.3**

Each value represents the mean ± standard deviation. * $p<0.05$, ** $p<0.01$, *** $p<0.001$ compared to the initial condition. GR: Glutathione reductase activity; GPx: Glutathione peroxidase activity; GSSG: Oxidized glutathione. GSH: Reduced glutathione. TBARs: Thiobarbituric acid reactive substances.
